# A Tool for Visualization and Analysis of Single-Cell RNA-Seq Data Based on Text Mining

**DOI:** 10.3389/fgene.2019.00734

**Published:** 2019-08-09

**Authors:** Gennaro Gambardella, Diego di Bernardo

**Affiliations:** ^1^University of Naples Federico II, Department of Chemical Materials and Industrial Engineering, Naples, Italy; ^2^Telethon Institute of Genetics and Medicine, Naples, Italy

**Keywords:** single-cell transcriptomics, term frequency–inverse document frequency, feature extraction, cell type, enrichment analysis

## Abstract

Gene expression in individual cells can now be measured for thousands of cells in a single experiment thanks to innovative sample-preparation and sequencing technologies. State-of-the-art computational pipelines for single-cell RNA-sequencing data, however, still employ computational methods that were developed for traditional bulk RNA-sequencing data, thus not accounting for the peculiarities of single-cell data, such as sparseness and zero-inflated counts. Here, we present a ready-to-use pipeline named *gf-icf* (gene frequency–inverse cell frequency) for normalization of raw counts, feature selection, and dimensionality reduction of scRNA-seq data for their visualization and subsequent analyses. Our work is based on a data transformation model named term frequency–inverse document frequency (TF-IDF), which has been extensively used in the field of text mining where extremely sparse and zero-inflated data are common. Using benchmark scRNA-seq datasets, we show that the *gf-icf* pipeline outperforms existing state-of-the-art methods in terms of improved visualization and ability to separate and distinguish different cell types.

## Introduction

Until very recently, the cost, time, and equipment needed to perform single-cell transcriptomics have limited their application to a few selected studies. Thanks to the new and cheap technologies (Klein et al., 2015; Macosko et al., 2015; Zheng et al., 2017), sequencing libraries for thousands of cells in a single experiment can now be prepared on a lab bench. Advanced computational approaches have been implemented to analyze these datasets and enabled discovery of new cell types (Butler et al., 2018; Aran et al., 2019) and the study of cellular dynamic processes at high temporal and spatial resolution (Trapnell et al., 2014; Achim et al., 2015; Satija et al., 2015; Liu and Trapnell, 2016). Moreover, single-cell RNA-sequencing (scRNA-seq) is reshaping our understanding of developmental biology, gene regulation, and cancer heterogeneity (Gawad et al., 2016). However, substantial computational obstacles remain because of the large amount of data and high levels of noise. Finding an effective low-dimensional representation of scRNA-seq data is the most important step for the data visualization and downstream analysis, such as cell type identification. At present, state-of-the-art computational pipelines for scRNA-seq data visualization consist in four main steps (Trapnell et al., 2014; Klein et al., 2015; Macosko et al., 2015; Shekhar et al., 2016; Zheng et al., 2017; Butler et al., 2018): i) normalizations of raw counts scaled by a sample-specific size factors; ii) feature selection by identifying most variable genes across cells; iii) dimensionality reduction with principal component analysis (PCA); and iv) projection of scRNA-seq data in an embedded space [such as t-SNE or UMAP tools (van der Maaten and Hinton, 2008; McInnes and Healy, 2018)]. Most steps in these pipelines, however, still employ computational methods that were developed for traditional bulk RNA-seq data, thus not accounting for the high level of noise caused by dropouts, leading to an excess of zeros and near-zero counts in the dataset.

Here, we present a ready-to-use pipeline named *gf-icf* (gene frequency–inverse cell frequency) for normalization of raw counts, feature selection, and dimensionality reduction (steps i to iii) whose results can be fed to t-SNE or UMAP for visualization of scRNA-seq data. *gf-icf* is based on a data transformation model called term frequency–inverse document frequency (TF-IDF), which has been extensively used in the field of text mining, where sparse and zero-inflated data are common (Robertson and Jones, 1976; Leskovec et al., 2014). Here, we show that the *gf-icf* pipeline outperforms the existing state-of-the-art methods exploiting a benchmark dataset of real cell mixture of FACS sorted cells (Zheng et al., 2017). We also show how features (i.e., genes) extracted from *gf-icf* can be used to automatically predict cell types outperforming methods based on top expressed genes.

## Methods

### Term Frequency–Inverse Document Frequency

In information retrieval or text mining, the term frequency–inverse document frequency (TF-IDF) is a data transformation and scoring scheme used for measuring the occurrences of a given word in a large collection of text documents (Robertson and Jones, 1976; Leskovec et al., 2014). Given a corpus of *N* documents, let *f_ij_* be the number of occurrence of the word *i* in the document *j*, and the term frequency *TF_ij_* of word *i* in the document *j* can be defined as: $TF_{ij}=f_{ij}/\sum_{}^{}\begin{array}{l} W\\ k=1 \end{array}f_{kj}$, where *W* is the number of words in document *j*. Hence, the term frequency of word *i* in document *j* represents its number of occurrences divided by the total number of occurrences of all the words in the same document. Thus, the sum of *TF* values of all the words in a document is always equal to 1. The inverse document frequency of word *i* can be instead defined as *IDF_i_* = log(*N*/*n*_i_+1), where *n_i_* denotes the number documents that contain word *i* out of the *N* documents in the corpus. Intuitively, the IDF value is high for a rare word and low for a common word. The TF-IDF score for word *i* in document *j* is simply $TF_{ij}\times IDF_{i}$. TF-IDF values of each document are then rescaled to have Euclidean norm equal to one (L2 normalization) to account for document length biases.

### Gene Frequency–Inverse Cell Frequency

Thanks to 3′-end scRNA-seq approaches, we can now have an accurate estimation of gene expression without having to account for gene length; thus, the number of transcripts (i.e., UMI) associated with each gene strictly reflects the frequency of a gene in a cell, exactly like a word in a document. Hence, we applied TF-IDF scores as defined above to scRNA-seq data considering a cell to be analogous to a document, genes analogous to words, and gene counts to be analogous of the word’s occurrence in a document. For the sake of clarity, we renamed in the manuscript TF-IDF to GF-ICF (gene frequency–inverse cell frequency). Moreover, since words with the highest TF-IDF score in a document are often the terms that best characterize the topic of that document, genes with the highest GF-ICF scores in a cell are expected to provide most information about the cell identity.

### t-SNE Visualization

After data normalization (GF-ICF or Seurat tool), the first 50 principal component were used as meta-genes to perform t-distributed stochastic neighbor embedding (t-SNE). t-SNE was run using Rtsne package in the R environment version 3.5.2. For t-SNE, we always used the same seed (equal to 0), the same value of perplexity equal to 30, and the same number of PCA components for all the analysis in order to improve replicability and comparison of tested methods. t-SNE coordinates were rescaled at [−1, 1] before plotting and computation of Euclidian distances among cells of the same type.

### Public Single-Cell Transcriptional Dataset

The single-cell transcriptional profiles of human peripheral blood mononuclear cells (PBMCs) of 10 distinct cell types identified by FACS analysis (Zheng et al., 2017) used in this study were directly downloaded from the 10X website (https://support.10xgenomics.com). Data were preprocessed to remove low-quality cells. Specifically, cells for which less than 500 genes and less than 1,500 UMI (unique molecular identifiers) were measured and for which the fraction of mapped mitochondria reads was higher than 10% were filtered out. After cell filtering, a total of 39,200 cells were retained and used for all downstream analyses. The 27,499 single-cell transcriptional profile from mouse retinal bipolar neurons (Shekhar et al., 2016) were obtained from GEO database (GSE81904). The single-cell transcriptional profiles of Tabula Muris project (Schaum et al., 2018) were obtained from *TabulaMuris* package of R statistical environment. Only the 55,656 cells that passed a quality control cutoff of 500 genes and 1,000 UMIs were used.

### Single-Cell Data Visualization With Seurat Tool

Seurat tool (v2) was used following the tutorial present on the Seurat website (https://satijalab.org/seurat). Briefly, raw counts were first normalized with *NormalizeData* function; then the most variable genes across the cells were identified using *FindVariableGenes* function. After UMI counts were rescaled with *ScaleData* function, principal component analysis (PCA) was performed using *RunPCA* function, and the top 50 PCA component were used for t-SNE visualization (*runTSNE* function) with value of perplexity equal to 30. t-SNE visualization and coordinate rescaling were performed as described above. All analyses were performed using R statistical environment version 3.5.2.

### Single-Cell Clustering and Relevant Gene Identification

Single-cell transcriptional profiles were normalized using the *gf-icf* method and projected with t-SNE in an embedded bi-dimensional space as described above. Cells were then clustered using a PhenoGraph like approach (Levine et al., 2015). From t-SNE coordinates, we first created a network of similar cells by calculating the Jaccard coefficient between the 50 nearest neighbors of each cell (using Manhattan distance), and then we identified communities in this network of cells using the Louvain method (Blondel et al., 2008).

### Cell Type Prediction

To predict cell type in each of the clusters, we extracted from each cluster its gene signature by summing their *gf-icf* scores across cells of the same cluster and selecting the top 100 genes with highest scores. We then performed gene set enrichment analysis (GSEA) (Subramanian et al., 2005) against a set of bulk transcriptomic data of “pure” cell types from a published study (Aran et al., 2019). Specifically, we used as a reference dataset the Blueprint Epigenomics dataset composed of 144 RNA-seq across 28 cell types (Stunnenberg et al., 2016) and the Encode dataset composed of 115 RNA-seq of pure stroma and immune samples across 17 cell types (Consortium et al., 2012) for a total of 45 distinct cell types. Finally, the top enriched cell type from GSEA was used to assign a cell type to each cluster.

### Adjusted Rand Index

The adjusted Rand index (ARI) proposed by Hubert and Arabie on *Journal of Classification* in 1985 (Hubert and Arabie, 1985) is the corrected-for-chance version of the Rand index (Rand, 1971) ARI is the most used index to evaluate the performance of a cluster algorithm when cluster’s labels are known a priori. It has the maximum value of 1, while its expected value is 0 in the case of random clusters. In this work, the ARI was computed using the function *adjustedRandIndex* of package *mclust* in the R statistical environments.

### Cluster Purity

Purity is an evaluation criterion of cluster quality that can be interpreted as the pureness of the final clusters compared with the classes of the ground truth (Hassani and Seidl, 2017). Purity was computed as follows: For each cluster of cells, we counted the number of cells from the most common cell type and divided it by the total number of cells across all the clusters. Formally, $Purity=\frac{1}{N}\sum_{}\begin{array}{l} m\in M \end{array}\underset{d\in D}{\max}|m\cap d|$, where *M* is the number of clusters, *D* a set of classes (i.e., cell types), and *N* the total number of cells.

## Results

### Identify-Relevant Genes Across Cell Populations

**Figure 1:**
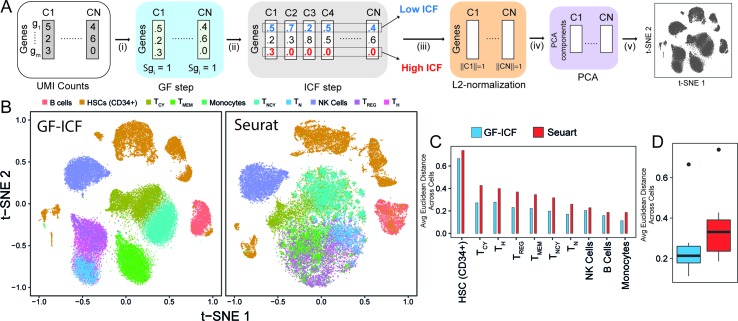
GF-ICF improves visualization of single-cell RNA-sequencing data. **(A)** The gf-icf pipeline. Starting from transcriptional profiles of a set of cells C1…CN, the pipeline consists of the following steps: (i) normalization of gene expression profiles of each cell to sum one (GF step); (ii) cross-cell normalization, to score rarely expressed genes higher than commonly expressed genes (ICF step); (iii) L2 normalization on each cell to obtain normalized gf-icf weights; and (iv) principal component analysis (PCA) to reduce the number of features (genes) dimensions before (v) projecting cell in an embedded space. **(B)** Comparison between t-SNE projection following gf-icf pipeline (left) and the Seurat tool (right) on 40k human PBMCs single-cell transcriptional profiles. Cells are colored according to their cell type of origin identified by FACS analysis by Grace et al. **(C)** Average Euclidean distance among PBMCs of the same type using either gf-icf pipeline or the Seurat tool. **(D)** Distribution of the average Euclidean distance among PBMCs of the same type using either gf-icf pipeline or the Seurat tool. Legend: T_CY_, cytotoxic T-cells; T_H_, helper T-cells; T_REG_, regulatory T-cells; T_MEM_, memory T-cells; T_NCY_, naïve cytotoxic T-cells; T_N_, naïve T-cells; NK cells, natural killer cells.

We aimed at developing a computational tool that could integrate single-cell transcriptional profiles across multiple conditions by extracting relevant genes to improve data visualization and cell type identification. The term frequency–inverse document frequency (i.e., TF-IDF) approach is a well-known statistical method to extract and select document-specific words (i.e., genes) across a large collection of documents (i.e., cells). The intuition behind the use of the TF-IDF approach to scRNA-seq data is that if a gene is highly expressed in a cell, it should be scored highly than less expressed genes in the same cell, but at the same time, highly expressed genes common to many cells of different types should be scored lower than genes expressed in a specific subpopulation of cells. As the TF-IDF approach has been extensively used in the context of text mining for feature selection and extraction (Robertson and Jones, 1976; Leskovec et al., 2014), we reasoned that this approach could be extremely useful to improve single-cell data analysis. Here, we developed the *gf-icf* (gene frequency–inverse cell frequency) pipeline, which is based on the TF-IDF approach, as schematized in **Figure 1A** (Methods). Briefly, given the transcriptional profiles of a set of cells *C_1_,*...*C_n_*, the pipeline consists of the following steps: i) normalization of gene expression profiles of each cell to sum one (GF step), thus removing bias related to cell coverage depth; ii) cross-cell normalization, to score rarely expressed genes higher than commonly expressed genes (ICF step) across subpopulations of cells (Methods); iii) L2 normalization on each cell to obtain normalized gf-icf weights; and iv) principal component analysis (PCA) to reduce the number of features (genes) dimensions before (v) applying t-SNE and project cells in a two-dimensional space.

### The *gf-icf* Pipeline Improves Cell Population Visualization and Clustering

We applied our *gf-icf* pipeline to analyze a published study of 39,200 human peripheral blood mononuclear cells (PBMCs) sequenced using the 10x Chromium platform and belonging to 10 distinct immune cell populations identified by cytofluorimetry (Zheng et al., 2017). After rescaling of t-SNE coordinates, we compared the visualization obtained from *gf-icf* to the state-of-the art method Seurat tool. As shown in **Figure 1B**, our strategy was able to improve single-cell data visualization by better separation of distinct cell types when compared with Seurat. Indeed, the only overlapping cell types were the CD4+ regulatory, CD4+ naïve, and CD4+ helper T-cells, which are cells derived from the same lineage (Glimcher and Murphy, 2000). To quantify and compare cell type separation between the two methods, we computed the average Euclidean distance of rescaled t-SNE coordinate among cells of the same cell type (**Figures 1C, D**). The same analysis was repeated using also rescaled UMAP (McInnes and Healy, 2018) coordinates (**Supplementary Figure 1**). We also applied our *gf-icf* pipeline to analyze an additional dataset consisting of 27,499 single-cell transcriptional profile from mouse retinal bipolar neurons (Shekhar et al., 2016) profiled using the Drop-seq platform. As shown in **Supplementary Figure 2**, our strategy was again able to improve single-cell data visualization when compared with Seurat. These results show that the *gf-icf* strategy can be successfully used to better visualize and separate distinct cell types. To further demonstrate how our approach can also enhance clustering of scRNA-seq data, we applied the *gf-icf* pipeline to the tabulaMuris data (Schaum et al., 2018) consisting of 55,656 single-cell transcriptional profile from 13 distinct mouse organs profiled using the Drop-seq platform. After the application of *gf-icf* normalization pipeline for normalization, visualization, and clustering of single-cell data (**Supplementary Figure 3**), we evaluated the purity of identified cluster by comparing it with cell types reported in the original publication. Specifically, we obtained a cluster purity of 83% and an adjusted Rand index of 0.51 in agreement with original publication analysis that was performed using Seurat tool (**Supplementary Figure 4**).

### *gf-icf* Normalization Selects and Extracts Relevant Genes for Cell Type Identification

**Figure 2:**
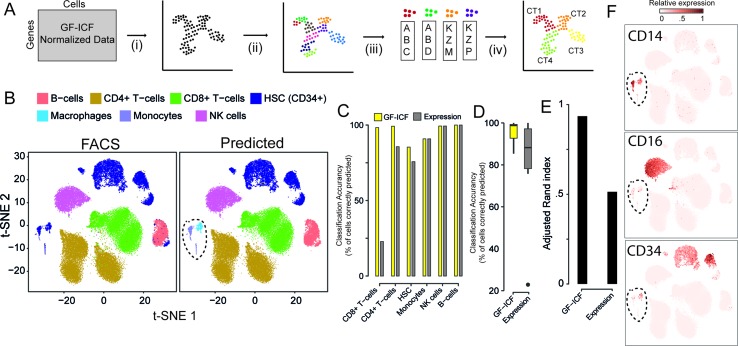
Relevant genes extracted from the *gf-icf* pipeline enable cell type prediction. **(A)** Pipeline for the identification of cell type using gf-icf pipeline. Single-cell transcriptional profiles are normalized by gf-icf in order to score genes in each single-cell, and then (i) cells are projected with t-SNE in a bi-dimensional space; (ii) cells are divided in small groups using Louvain–Jaccard clustering; and (iii) the gene signature of each cluster is identified and (iv) used to predict cell type of origin by gene set enrichment analysis (GSEA) against a set bulk transcriptomic data of pure cell types. **(B)** Comparison between FACS-sorted cell type (left) and predicted cell type (right) of about 40k PBMCs. **(C)** Cell type prediction accuracy as a percentage of correctly predicted cells using either gf-icf or normalized counts. **(D)** Distribution of cell type prediction accuracy using either gf-icf or normalized counts. **(E)** Adjusted Rand index of cell type prediction using either gf-icf or normalized counts. **(F)** Expression of CD14, CD16, and CD34 marker genes for a small subpopulation of HSCs predicted instead to be monocyte and macrophages.

We next asked whether relevant genes identified by *gf-icf* normalization are better biomarkers than the ones selected simply using normalized counts, as currently done in standard scRNA-seq pipelines. We first validated our approach on the 39,200 PBMCs cells for which cell type of origin had been identified by cytofluorimetric analysis (Zheng et al., 2017). For simplicity, we grouped immunological cell types into six main types: 1) CD4+ cells, 2) CD8+ cells, 3) B-cells, 4) natural killer (NK) cells, 5) homeopathic stem cells (HSCs, CD34+), and 6) monocyte. We applied the pipeline described in **Figure 2A**: starting from scRNA-seq data processed with the *gf-icf* method, cells are projected with t-SNE in an embedded bi-dimensional space, and then i) cells are clustered into transcriptional homogenous clusters; ii) the top 100 genes with the largest *gf-icf* scores represent the gene signature of each cluster (Methods); iii) gene set enrichment analysis (GSEA) is performed using as a set the gene signature of a cluster and as a reference the bulk transcriptional profiles of cytofluorimetry-sorted cell types derived from the Encode and Blueprint Epigenomics databases (Consortium et al., 2012; Stunnenberg et al., 2016); and iv) in the last step, the inferred cell types for each cluster are visualized using a color code. As shown in **Figure 2B**, we correctly assigned the majority of cells to their cell type of origin, achieving an average accuracy of cell type classification of 96% (**Figures 2C, D**) and an adjusted Rand index (Hubert and Arabie, 1985) of 0.94 (**Figure 2E**). We then repeated the above analysis but this time using as gene signatures the 100 genes most expressed in each cluster, rather than the 100 genes with the largest gf*-icf* scores. In this case, we achieved a lower average cell classification accuracy of 79% (**Figures 2C, D** and **Supplementary Figure 5**) and a lower adjusted Rand index of 0.52 (**Figure 2E**). These results show that the *gf-icf *strategy can be successfully used to improve feature selection and to identify-relevant genes in distinct cell populations. Interestingly, as shown in **Figure 2B** (dashed circle), a small group of cells, which according to cytofluorimetry were classified as hemopoietic stem cells, was predicted by our analysis to consist of monocytes and macrophages. Expression of the canonical monocyte and macrophage markers (i.e., CD14 and CD16) and lack of CD34 expression seem to confirm our predictions (**Figure 2F**).

## Discussion

Single-cell RNA-seq is now the technology of choice to identify the different cell types composing the human body and to elucidate embryo development. However, computational methods for dimensional reduction, visualization, and clustering of scRNA-seq data still remain challenging. Finding an effective low-dimensional representation of single-cell data is a key step for visualization and subsequent analyses. For example, such representations can be used to detect “good” clusters across the profiled set of cells, thus greatly improving the identification of biomarker genes, which are often identified from differentially expressed genes across the clusters (Trapnell et al., 2014; Zheng et al., 2017). Moreover, co-expression analysis can be performed across clusters (Gambardella et al., 2017) in order to identify differentially co-expressed set of genes (Gambardella et al., 2013, Gambardella et al., 2015) and thus predict active gene regulatory networks.

Here, we developed an accurate and efficient ready-to-use pipeline named *gf-icf* (https://github.com/dibbelab/gficf), which provides an effective and simple workflow for the normalization of raw counts, feature selection, and dimensionality reduction whose results can be fed to t-SNA or UMAP for visualization and analysis of single-cell data. *gf-icf* is based on a well-established data transformation called TF-IDF. Recently, this technique has indeed been shown to improve scRNA-seq data clustering (Moussa and Mandoiu, 2018). Here, we improve previous results by taking into account differences in the number of reads by using Euclidian normalization and extend the use of TF-IDF to improve data visualization. Moreover, we implemented a ready-to-use pipeline in R to make this technique available to anyone. Empirical evaluation of the *gf-icf* pipeline on a real cell mixture of FACS sorted cells consistently outperformed existing state-of-the-art pipelines.

## Data Availability

R package of *gf-icf* pipeline and examples of use are available at the following address: https://github.com/dibbelab/gficf

## Author Contributions

GG conceived and developed the tool, while DB supervised the work and contributed to the writing the manuscript.

## Funding

This work was supported by the STAR (Sostegno Territoriale alle Attività di Ricerca) grant of University of Naples Federico II to GG and by the AIRC (Associazione Italiana Ricerca sul Cancro) Grant IG 2016-18479 to DB.

## Conflict of Interest Statement

The authors declare that the research was conducted in the absence of any commercial or financial relationships that could be construed as a potential conflict of interest.
