# Supplementary material for: A Tool for Visualization and Analysis of Single-Cell RNA-Seq Data Based on Text Mining
Source: Front Genet. 2019 Aug 9;10:734. doi: 10.3389/fgene.2019.00734 (PMC6696874; doi:10.3389/fgene.2019.00734)
Supplement: Supplementary file 1 [file DataSheet_1.pdf]

Supplementary Material

1 Supplementary Figures

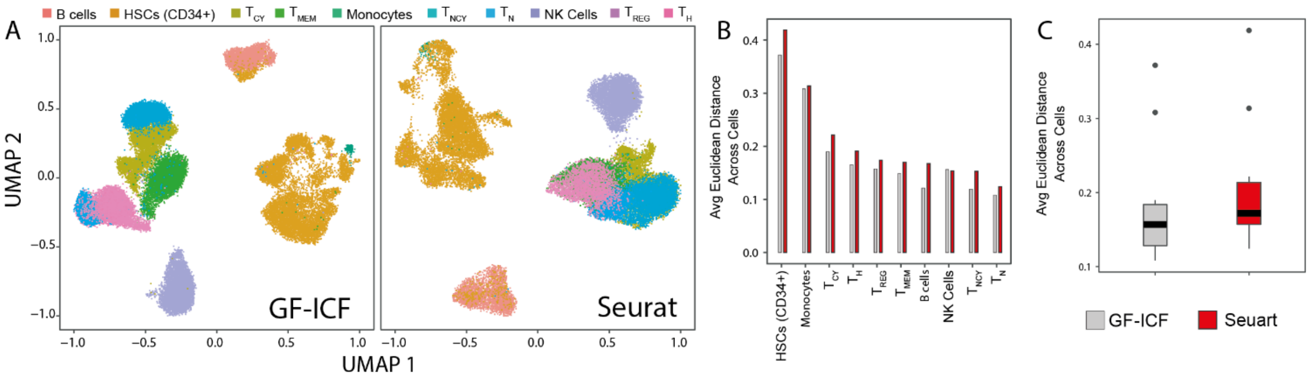

Supplementary Figure 1 – gf-icf improves cell clustering and visualization (A) Comparison between UMAP (McInnes et al., 2018) projection of about 40k human PBMCs single cell transcriptional profiles (Material and Methods) after have been normalized with gf-icf pipeline (left) or Seurat tool (right). Cells are coloured according to their cell type of origin identified by FACS analysis by Grace et al. (**B**) Average Euclidean distance among PBMCs of the same type using either gf-icf pipeline or the Seurat tool (**C**) Distribution of the average Euclidean distance among PBMCs of the same type using either gf-icf pipeline or the Seurat tool. Legend: T_{CY}=Cytotoxic T-cells; T_H=Helper T-cells; T_{REG}=Regulatory T-cells; T_{MEM}=Memory T-Cells; T_{NCY}=Naïve Cytotoxic T-Cells; T_N=Naïve T-Cells; NK Cells=Natural Killer Cells

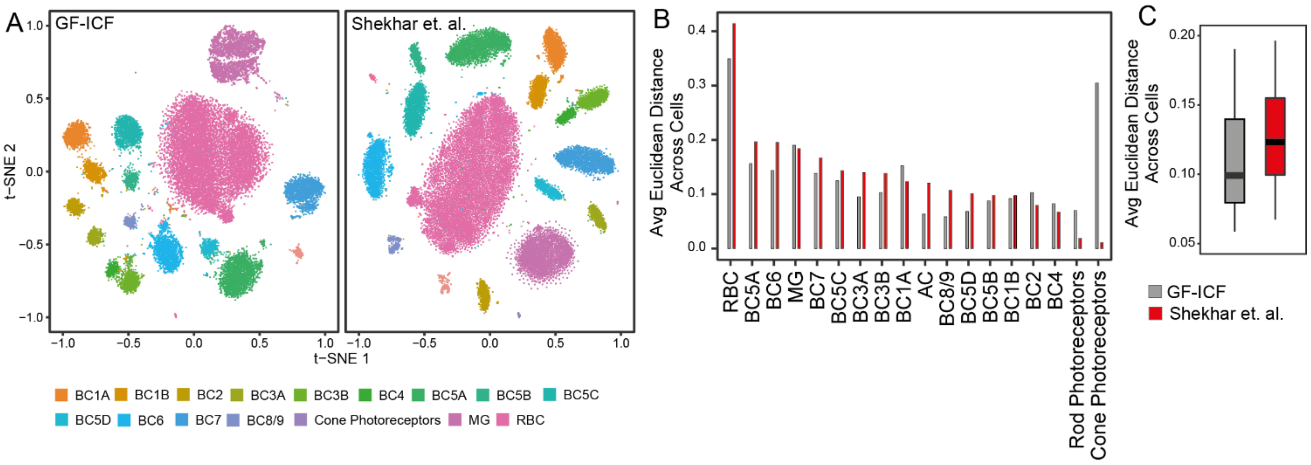

Supplementary Figure 2 - gf-icf improves cell clustering and visualization (A) Comparison between t-SNE (Laurens van der Maaten and Hinton, 2008) projection of about 27k mouse retina bipolar single cell transcriptional profiles after have been normalized with gf-icf pipeline (left) or as reported in the original manuscript (Shekhar et al., 2016) (right). Cells are coloured according to their cell type of origin as identified in the original manuscript (Shekhar et al., 2016) (**B**) Average Euclidean

distance among retinal bipolar cells of the same type using either gf-icf pipeline or the rescaled coordinated of t-SNE projection in the original publication (Shekhar et al., 2016) (**C**) Distribution of the average Euclidean distance among retinal bipolar cells of the same type using either gf-icf pipeline or the rescaled coordinated of t-SNE projection in the original publication (Shekhar et al., 2016).

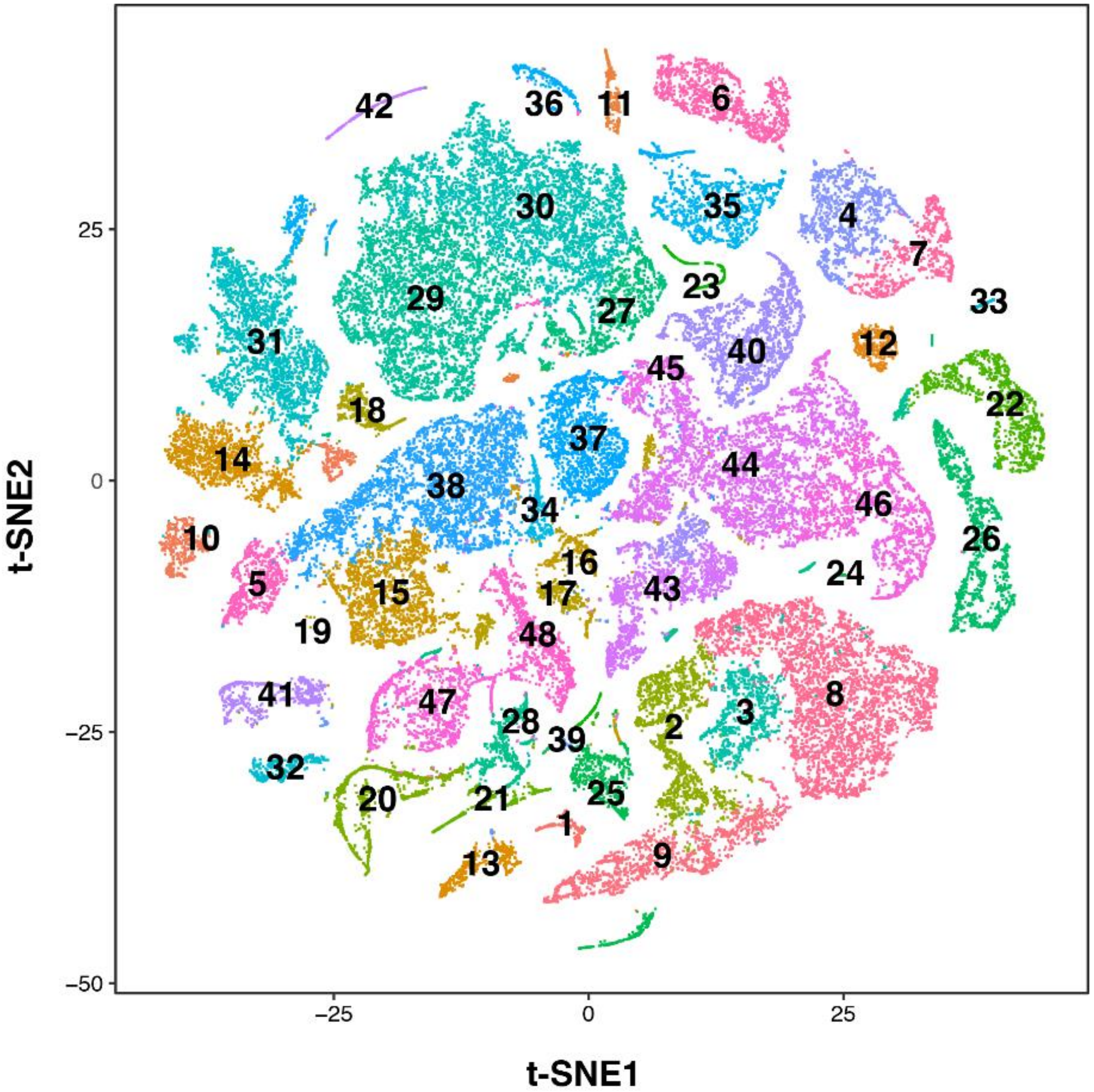

Supplementary Figure 3 – t-SNE projection of 55,656 mouse cells from TabulaMuris project (Schaum et al., 2018) after having been normalized with gf-icf pipeline. Cells were clustered using Louvain and colored according to the cluster they belong. Clusters were numbered from 1 to 48.

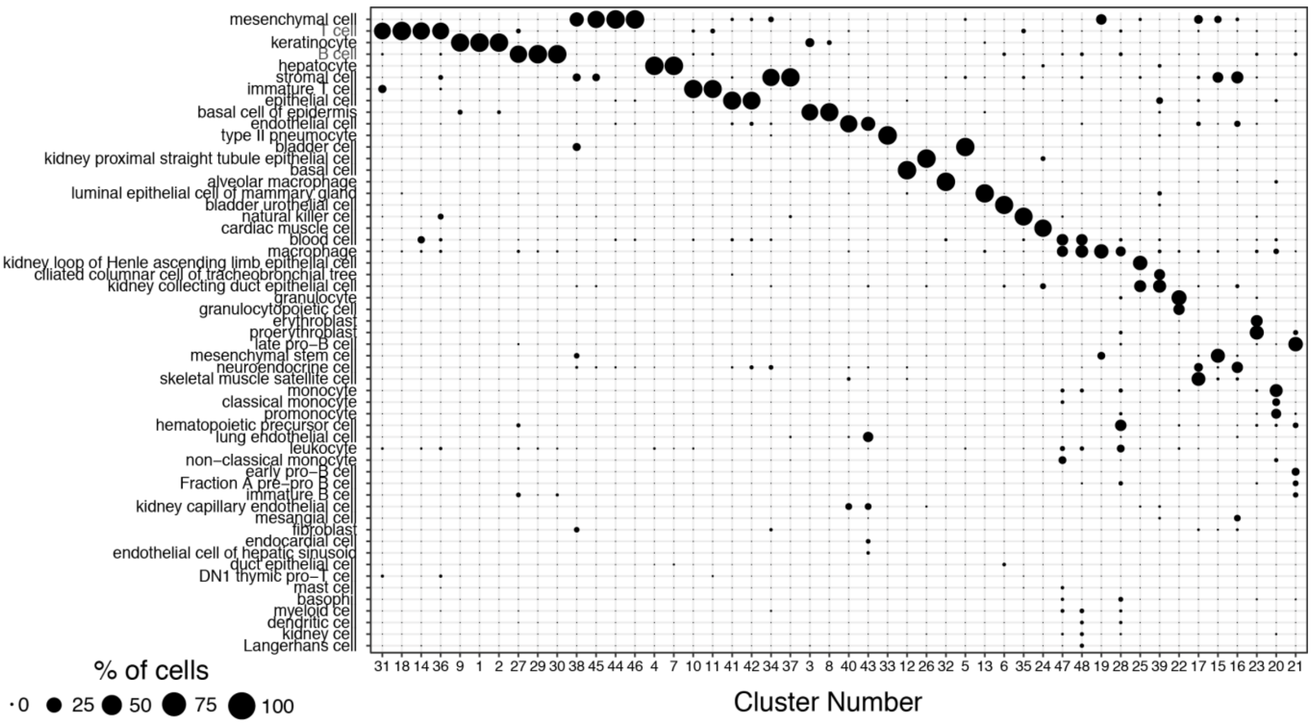

Supplementary Figure 4 – Cluster Purity. For each of the 48 clusters we identified in TabulaMuris data its cell type composition is reported. Cell type of each cell was retrieved from the original publication (Schaum et al., 2018).

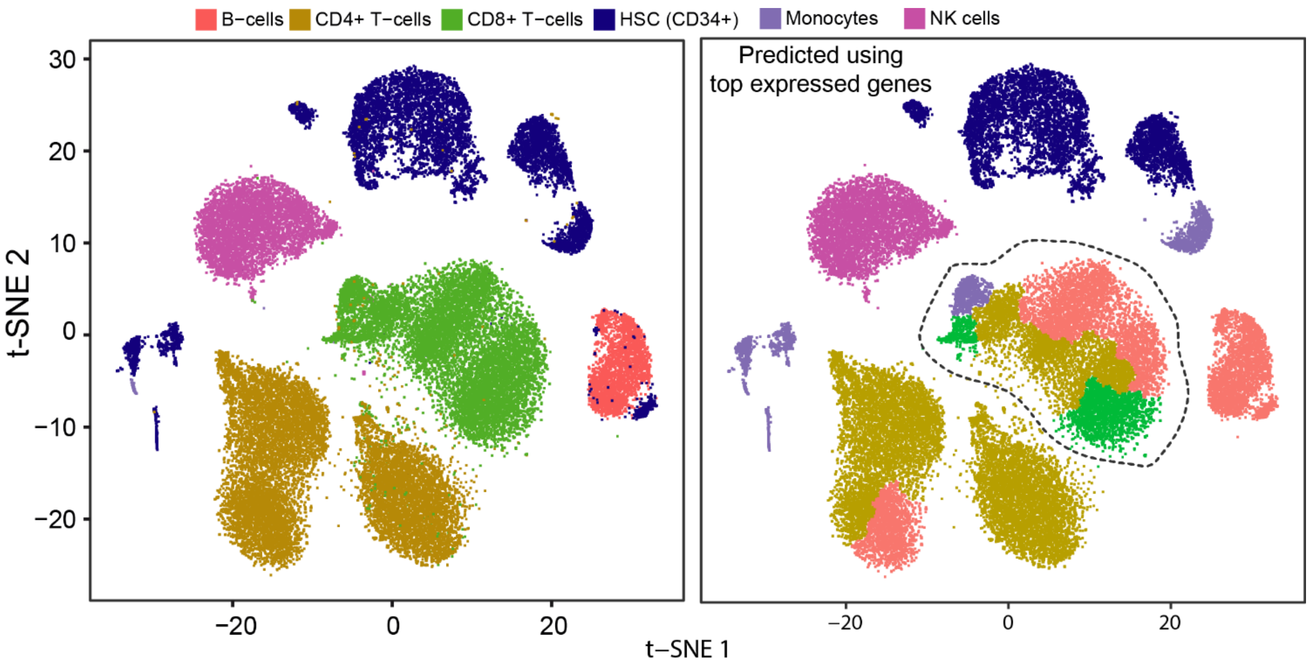

Supplementary Figure 5 – Cell type prediction using top expressed genes. Comparison between FACS-sorted cell type (left) and predicted cell type (right) using top expressed genes instead of gf-icf

scoring for about 40k human PBMCs (Material and Methods). CD8+ T-cell (dotted circle) are almost all predicted wrong.

2 References

Laurens van der Maaten, and Hinton, G. (2008). Visualizing Data using t-SNE. J. Mach. Learn. Res. *9*, 2579–2605.

McInnes, L., Healy, J., and Melville, J. (2018). UMAP: Uniform Manifold Approximation and Projection for Dimension Reduction.

Schaum, N., Karkanias, J., Neff, N.F., May, A.P., Quake, S.R., Wyss-Coray, T., Darmanis, S., Batson, J., Botvinnik, O., Chen, M.B., et al. (2018). Single-cell transcriptomics of 20 mouse organs creates a Tabula Muris. Nature *562*, 367–372.

Shekhar, K., Lapan, S.W., Whitney, I.E., Tran, N.M., Macosko, E.Z., Kowalczyk, M., Adiconis, X., Levin, J.Z., Nemesh, J., Goldman, M., et al. (2016). Comprehensive Classification of Retinal Bipolar Neurons by Single-Cell Transcriptomics. Cell *166*, 1308-1323.e30.
